# A Review on Curcumin-Loaded Electrospun Nanofibers and their Application in Modern Medicine

**DOI:** 10.1007/s11837-022-05180-9

**Published:** 2022-02-24

**Authors:** Souradeep Mitra, Tarun Mateti, Seeram Ramakrishna, Anindita Laha

**Affiliations:** 1 Department of Chemical Engineering, Manipal Institute of Technology, Manipal Academy of Higher Education, Manipal - 576104 Udupi, Karnataka India; 2 Center of Nanofibers and Nanotechnology, National University of Singapore, Singapore, 117581 Singapore

## Abstract

Herbal drugs are safe and show significantly fewer side effects than their synthetic counterparts. Curcumin (an active ingredient primarily found in turmeric) shows therapeutic properties, but its commercial use as a medication is unrealized, because of doubts about its potency. The literature reveals that electrospun nanofibers show simplicity, efficiency, cost, and reproducibility compared to other fabricating techniques. Forcespinning is a new technique that minimizes limitations and provides additional advantages to electrospinning. Polymer-based nanofibers—whose advantages lie in stability, solubility, and drug storage—overcome problems related to drug delivery, like instability and hydrophobicity. Curcumin-loaded polymer nanofibers show potency in healing diabetic wounds in vitro and in vivo. The release profiles, cell viability, and proliferation assays substantiate their efficacy in bone tissue repair and drug delivery against lung, breast, colorectal, squamous, glioma, and endometrial cancer cells. This review mainly discusses how polymer nanofibers interact with curcumin and its medical efficacy.

## Introduction

Ayurveda is the oldest evidence of the use of medicinal plants which dates back approximately 5000 years, and inscribed within are drug preparations referring to 250 various herbs.^1^ Remedies made from herbs like Pushkarmool (*Inula racemosa*), Dhamanaka (*Artemisia nilagirica*), Pippali (*Piper longum*), Kalmegh (*Andrographis paniculata*), Bhumamalaki (*Phyllanthus amarus*), Tulsi (*Ocimum sanctum*), and Liquorice (*Glycyrrhiza glabra*) () treat disorders relating to anxiety,^2^ diabetes,^3^ cholesterol,^4^ and even cancer^5^ by improving the body's immunity.^6^ A study has snown that around 80% of the population of South Asian countries (Afghanistan, Bangladesh, Bhutan, India, Maldives, Nepal, Pakistan, and Sri Lanka) use herbal products in their daily practice.^7^ As a result, people preferred herbal drugs over allopathic ones during the SARS-CoV-2 (COVID-19) pandemic.^8-10^

Curcumin is an active ingredient found in turmeric,^11^ and benefits inflammatory conditions,^12^ metabolic syndrome,^13^ pain,^12^ and kidneys,^14^ and helps to manage inflammatory and degenerative eye conditions.^15^ However, curcumin is not a commercial medication because of its poor bioavailability and other intrinsic properties.^16^

**Fig. 1:**
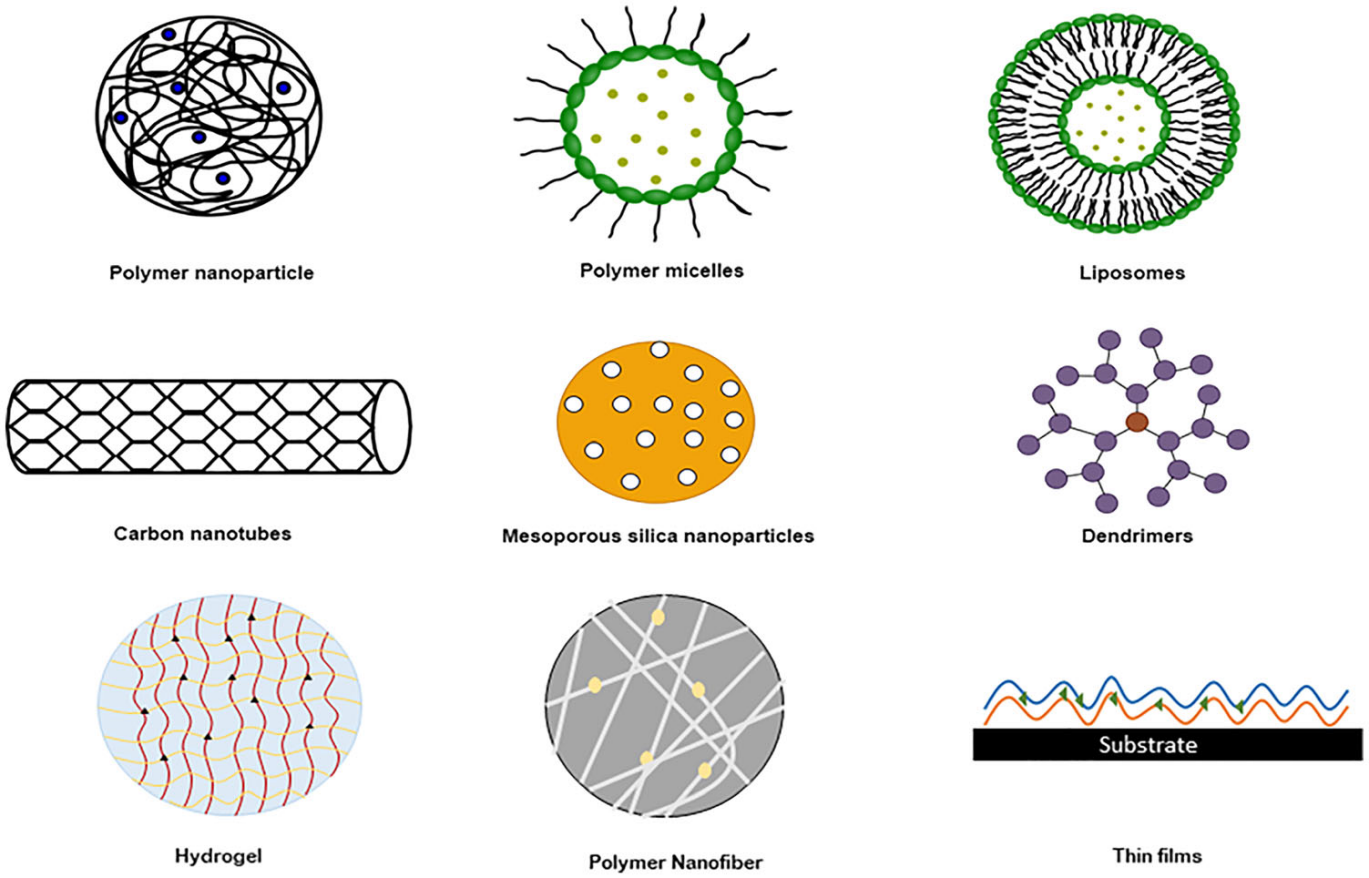
Nanocarriers for drug delivery.

Drug delivery involves incorporating a drug into a nanocarrier to deliver at a targeted location without adverse effects on other human body parts. Nanocarriers decrease the drug's toxicity and improves its efficacy.^17-22^ Incorporating curcumin into a suitable nanocarrier improves its bioavailability and efficacy by controlled and preferential drug release at the target site, and enhances its stability and solubility.^23^ Some examples of nanocarriers include polymer nanoparticles,^24^ micelles,^25^ liposomes,^26^ nanotubes,^27^ dendrimers,^28^ hydrogels,^29^ mesoporous silica nanoparticles,^30^ thin films,^31^ and nanofibers,^32^ as shown in Fig. 1*.*

While significant research on drug efficacy in various delivery vehicles is available, nanofibers are a better choice due to their high drug-encapsulating efficiency, high surface-area-to-volume ratio, and good cell adhesion and proliferation characteristics.^33^ There exist various physical and chemical methods to fabricate nanofibers, such as physical vapor deposition,^34^ laser ablation,^35^ chemical vapor deposition,^36^ electrochemical deposition,^37^ template-assisted synthesis,^38^ and electrospinning.^39-41^ Each method has its advantages, but electrospinning aids in the fabrication by its simplicity, low cost, and high efficiency.^42^ Such nanofibers provide control over the morphology and give an ease to functionalize respective to the application. Thus, electrospun nanofibers have an undoubted advantage over other drug delivery vehicles due to their cheap fabrication cost and ease of scalability, alongside all the mentioned advantages.

Forcespinning is a technique in which centrifugal force draws the fibers instead of an electric field, as used in the electrospinning technique. It minimizes many of the limitations of electrospinning and provides commercialization ease, improved production rates, increased material choice, and lower fiber cost.^43–45^ Despite similar properties to electrospinning and many advantages, forcespinning is very new in fabricating nanofibers for drug delivery purposes. A few significant differences between electrospinning and forcespinning techniques are mentioned in Table I.^43,45^

**Table I:** Differences Between Electrospinning and Forcespinning Techniques

Electrospinning technique	Forcespinning technique
Uses electrostatic force to draw fibers	Uses centrifugal force to draw fibers
Requires understanding of other parameters that affect Taylor cone and jet instability	Requires no such understanding
Fiber diameter depends on solution concentration, conductivity, viscosity, surface tension, applied voltage, feed rate, the distance between tip and collector, and environmental conditions	Fiber diameter depends upon rotational speed, spinneret selection, rheological properties, nozzle configuration, collection system and environmental conditions
Requires high electric field	Does not require any electric field
The solution used must be typically dielectric for nanofibers to be spun out	Both conductive and non-conductive solutions can be used for nanofibers to be spun out
Comparatively lower production rate	Comparatively higher production rate
Comparatively higher fiber production cost	Comparatively lower fiber production cost

Biomaterials are vital in understanding drug activity and delivery mechanisms using 3D in vitro tumor models.^46^ Numerous biomaterials—chitosan,^47^ bovine serum albumin,^48^ gelatin,^49–51^ zein,^52,53^ bombyx mori silk,^54^ PCL,^55-57^ PVA,^58-60^ and PLA^61-63^ among many—have been engineered for various biomedical purposes. Their sought for properties include biocompatibility, non-immunogenicity, solubility, and biodegradability, apart from their respective inherent properties, making them suitable for drug delivery applications. However, only a few biomaterials have been tested on humans, as pharmaceutical companies are wary of evaluating novel biomaterials that have never previously been used in medication manufacture, even when the cost of doing so is low.

The purpose of this review is to provide insights into the biomedical applications of curcumin-loaded nanofibers in various medical conditions, and to urge for its extensive commercial use.

## Curcumin: A Diamond in the Rough

**Fig. 2:**
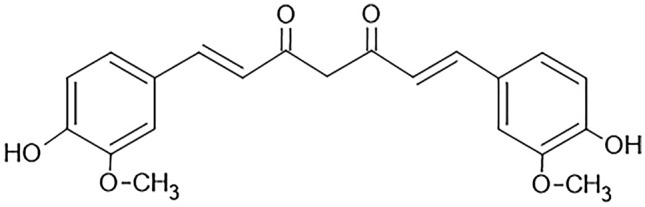
Chemical structure of curcumin.

**Fig. 3:**
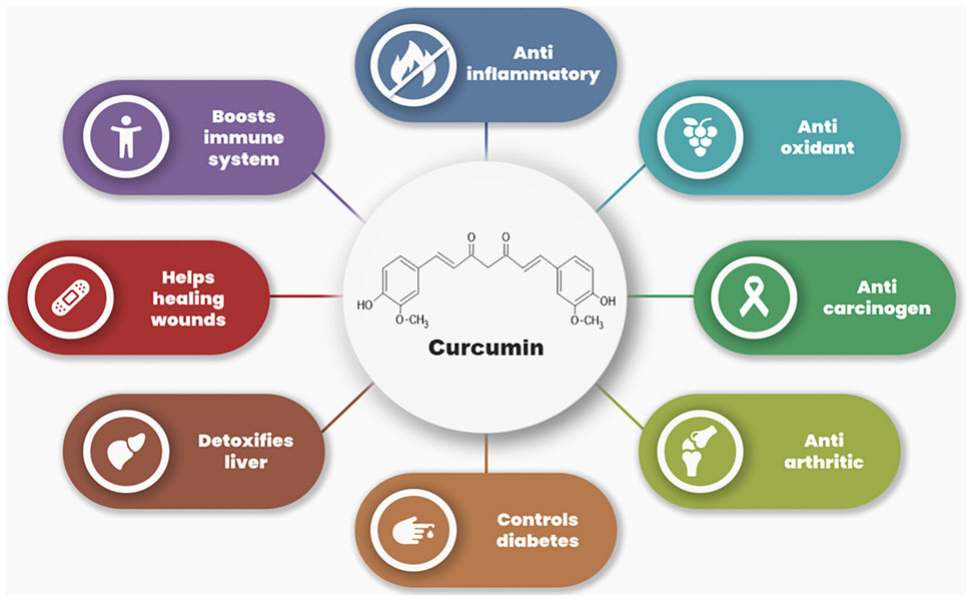
Benefits of curcumin.

Curcumin (Fig. 2)—the primary natural polyphenol found in the rhizome of *Curcuma longa* (turmeric) and other species—was extracted from turmeric in pure crystalline form for the first time in 1870.^64-66^ Asian countries traditionally use *Curcuma longa* as a medical herb for health benefits (Fig. 3). Curcumin targets molecules at the cellular level, and helps to improve inflammatory conditions, metabolic syndrome, pain, and eye conditions, and benefits the kidneys.^11^

Curcumin is at least ten times more active as an anti-oxidant than vitamin E.^67^ It shows its anti-oxidant effect by scavenging free radicals (reactive oxygen and nitrogen species), modulating enzymes that neutralize free radicals, and inhibiting such species-generating enzymes.^11^

Curcumin showed its anti-inflammatory property when tested on colorectal cells by hindering inflammatory receptors and associated proteins.^68^ Moreover, it impeded tumor-associated inflammation in most diseases.^11^

Curcumin also exhibits anticarcinogenic activities by inducing cell death through suppressing cell survival proteins,^69^ and anti-arthritic effects in osteoarthritis and rheumatoid arthritis. Furthermore, a study has suggested curcumin as a painkiller.^70^

Curcumin attenuates several aspects of metabolic syndrome by improving insulin sensitivity, lowering hypertension, and inflammation. It may also reduce muscle soreness and anxiety in obese people.^11^

Despite numerous benefits, the major criticism regarding curcumin is its poor bioavailability due to poor absorptivity, high metabolic rate, chemical instability, and quick body rejection. Most curcumin ingested is excreted in the feces (~90%).^71-73^ Curcumin derivatives like tetrahydro curcumin or the curcumin–piperine complex could address these issues and enhance its bioavailability. Otherwise, nanotechnology could also increase its bioavailability.^16^

Encapsulating curcumin into nanocarriers is an appealing choice to increase its biological activity by increasiung its bioavailability and solubility, circulation duration, and retention in the body, and overcome its physiological barriers. To this end, researchers have shown the feasibility of using nanoformulation-based approaches involving liposomes,^74^ polymer conjugates,^74^ cyclodextrins,^75^ micelles,^76^ and nanoparticles.^77^

The approach of interest is the use of nanofibers to enhance its bioavailability. Nanofibers are fibers with diameters in the nanometer range, providing good encapsulation, and their high surface-area-to-volume ratio combined with a microporous structure favors the cell adhesion, proliferation, migration, and differentiation desired for drug delivery and tissue engineering.^33^^,^^78^

## Electrospinning Technique

Nanofibers can be synthesized from various polymers and have varying physical properties. Some examples include poly(lactic acid) (PLA), polyurethane (PU), and poly(3-hydroxybutyrate-co-3-hydroxyvalerate) (PHBV).^79^ The diameter depends upon the polymer and the production method. Many chemical and mechanical techniques exist for their preparation, such as electrospinning, thermal-induced phase separation, drawing, template synthesis, and self-assembly. While each method has its benefits and liabilities, we are keen to discuss the electrospinning method because of its simplicity, high efficiency, low cost, and high reproducibility compared to others. It provides flexibility in choosing the preparation materials, making them potent in biomedical applications.^42,80^

**Fig. 4:**
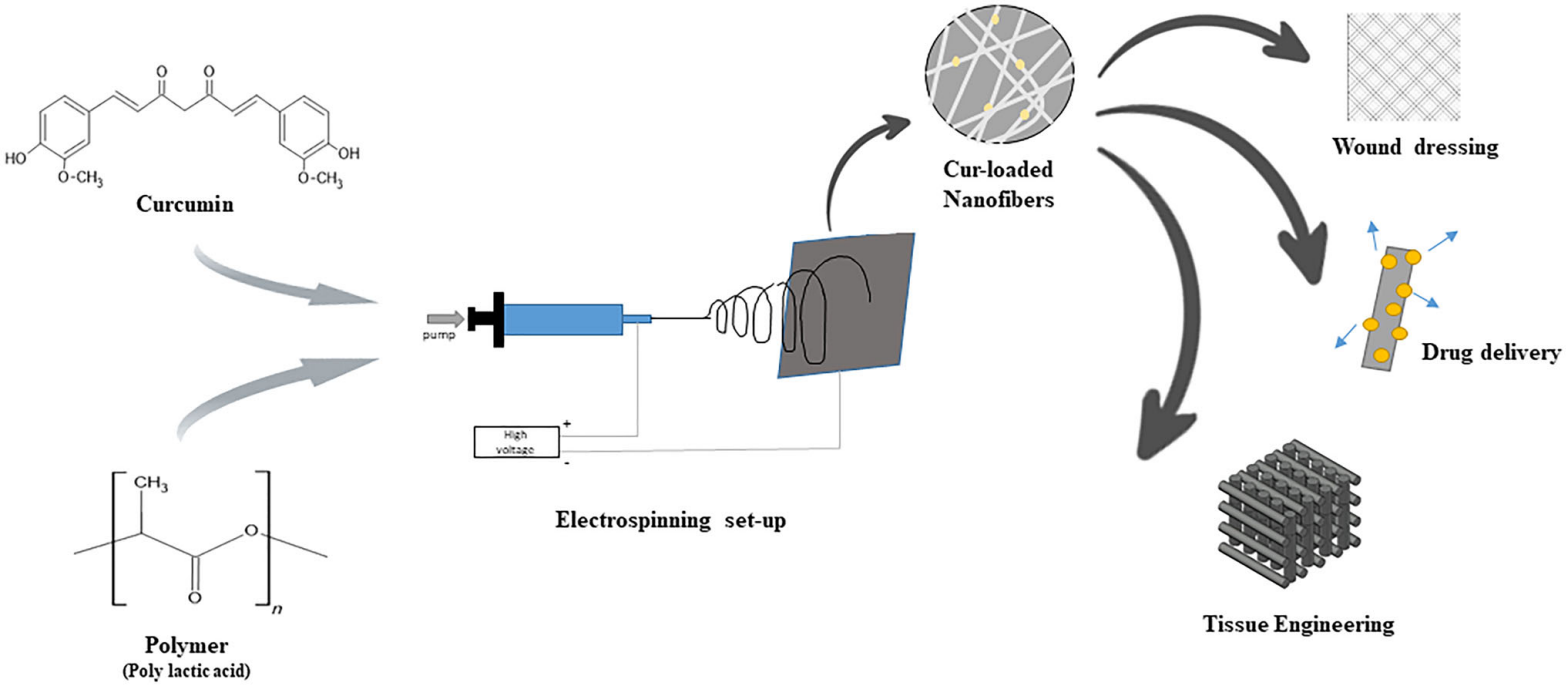
Electrospinning technique of nanofibers.

Electrospinning (Fig. 4) is a fiber production method that uses electric force to draw charged threads of polymer solutions or melts to a diameter in nanometers. Cooley patented it in May 1900 and February 1902,^81^ and W.J.Morton in July 1902. However, electrospinning was first used to produce fiber in 1934 by Anton Formhals.^82^

The setup of the electrospinning process involves:

- A power source
- A metallic needle
- Ground collector

### Working principle

A pendant droplet of polymer forms when an electrostatic force (from a power source) is applied to a polymer solution. When the force overcomes the fluid's surface tension, a Taylor cone forms at the needle's tip by deforming the pendant droplet, and when the force exceeds the conical droplet's surface tension, a jet of the polymer solution ejects from the needle's tip into long, thin filaments that solidify and then deposited on a grounded collector, forming uniform nanofibers.^83^

The characteristics of the electrospun nanofibers depend upon many parameters. These parameters are as follows:

### Solution Parameters

(a) Solution concentration, on which the nanofiber diameter depends. By reducing the concentration of the polymer solution, fibers with smaller diameters can be produced.^84^ (b) The electric conductivity relates to the nanofiber diameter: increasing the electric conductivity reduces the nanofiber diameter. (c) Viscosity primarily determines the diameter and morphology of the nanofibers. Viscous polymer solutions lead to uniform and larger fibers.^85^ (d) For initiation of the electrospinning process, the charged solution needs to overcome the solution's surface tension. Solvents with less surface tension favor smooth fiber formation.

### Process Parameters

(a) Applied voltage affects the nanofiber diameter, as fiber formation occurs when the applied voltage exceeds the threshold.^83^ (b) The feed rate of the solution: with an increase in feed rate, there is an increase in the fiber diameter. However, when the feed rate is too high, bead formation in the fiber occurs due to providing insufficient time for solvent evaporation.^83^ (c) A minimum distance between the tip and the collector needs to be determined for solvent evaporation before reaching the collector. Beads would occur if the distance were too far or too close.^86,87^

### Ambient Conditions

Temperature and humidity are essential to the quality of the nanofibers. Increasing the surrounding temperature from 25°C to 60°C resulted in a decreased fiber diameter caused by decreased viscosity. At very low humidity, the solvent evaporation rate increases, and the solvent dries very fast, while at high humidity, it leads to solution discharge.^83,88^

## Advancements in Curcumin Electrospun Nanofibers

**Fig. 5:**
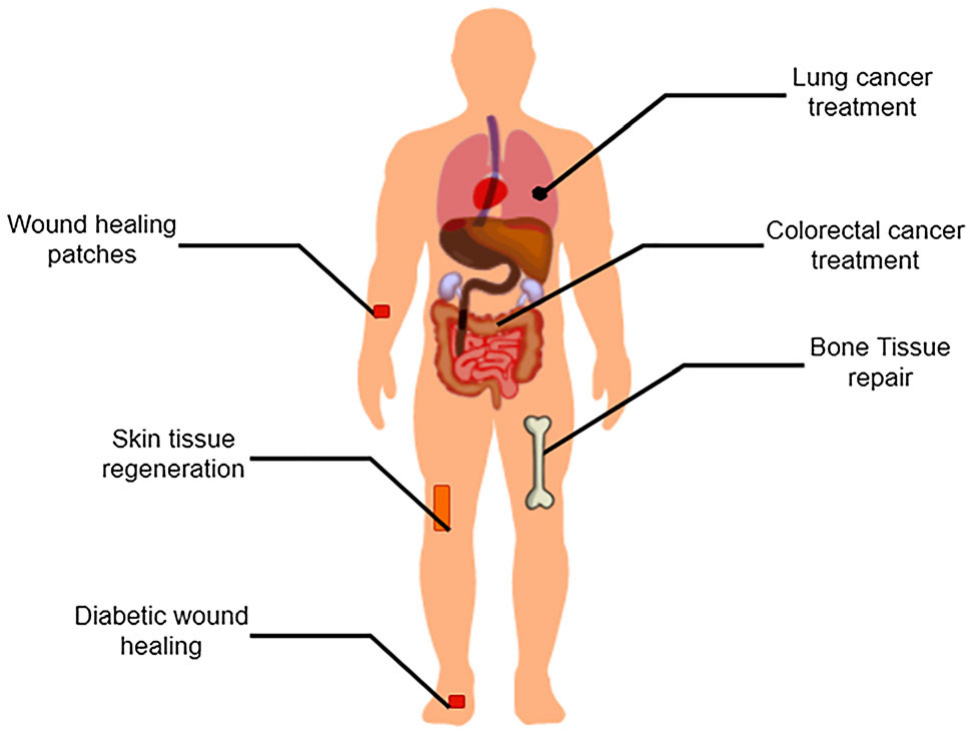
Applications of curcumin-loaded nanofibers.

Research exists on curcumin nanofibers usage in drug delivery (Fig. 5). PU, PHBV, poly(lactic-co-glycolic acid) (PLGA), *Bombyx mori* silk, and zein silk are some of the polymers used to synthesize nanofibers for medical purposes. However, the most commonly used polymers to generate nanofibers are poly(ε-caprolactone) (PCL), PLA, and polyvinyl alcohol (PVA) due to their intriguing properties.

*DCM* dichloromethane, *DMF* dimethylformamide, *IPA* isopropyl alcohol,*DMA* dimethylacetamide

### Poly(ε-Caprolactone) (PCL)

Resarchers have chosen PCL nanofibers (Table II) as a delivery vehicle^56,57^ due to their biocompatibilityand non-immunogenic and biodegradable nature. Merrell et al.^89^ performed the first substantial work on curcumin-loaded PCL nanofibers for diabetic wound dressings. Theys chose two different curcumin concentrations (3% w/w and 17% w/w), and a low polymer concentration (<14%) resulting in bead formation, which could be due to the low viscoelastic behavior of the polymer solution.^113^ Incorporating curcumin at 3% (w/w) led to a bead-free morphology and a broad diameter distribution (200–800 nm). The maximum amount of curcumin loaded under optimized conditions was 17%, which resulted in its precipitation from the solution on a further increase. The wound closure rate of 17% Ccrcumin fibers was higher than others. Cell viability of cultured cells (HFF-1) decreased with increased curcumin concentration, and the fibers demonstrated a sustained release pattern.^89^

**Table II:** Scientific evidence for potential applications of curcumin nanofibrous drug delivery

No.	Polymer	Drug(s)	Solvent		Remarks	Reference
1	PCL	Curcumin	Chloroform: Methanol (3:1 v/v)		In vitro and in vivo wound healing study in Streptozotocin diabetic mouse	^89^
2	PCL/PEG	Curcumin	DCM: DMF (90:10 v/v)		In vitro and in vivo wound healing study	^90^
3	PCL/Gum tragacanth	Curcumin	Acetic acid (90% v/v)		In vitro diabetic wound healing study	^91^
4	PCL/PEG	Tetrahydro Curcumin	Chloroform : Acetone (7:3)		In vitro study of wound healing patches	^92^
5	PCL/PEG	Curcumin/Chrysin	Acetone, chloroform, methanol (2:1.5:1.5) and DMSO		In vitro wound healing application	^93^
6	PCL/ [Carboxymethyl chitosan /PVA/GO]	Curcumin/Zn	DMF and DCM (1:3)		In vitro evaluation for bone tissue repair	^94^
7	PCL-PEG	Curcumin	DCM:IPA (4:1)		In vitro evaluation against glioma 9L cells	^95^
8	PCL	Curcumin/neem	Chloroform : Methanol (7:3)		In vitro evaluation against lungs and breast cancer cells	^96^
9	PCL/PEG	Curcumin	Chloroform : Methanol (4:1 v/v)		In vitro and in vivo evaluation of Endometriosis in mice model	^97^
10	PLA	Curcumin	Acetone and DMA (2:1 v/v)		In vitro study for evaluating potential biomedical applications	^98^
11	PLLA	Curcumin	Chloroform		In vitro study for potential anti-cancer drug delivery application	^99^
12	PLA	Curcumin	Chloroform and DMA (80:20 weight ratio)		In vitro and in vivo wound healing study	^100^
13	PLLA	Curcumin	DCM:DMF (7:3 v/v)		In vitro study for wound healing application	^101^
14	PLA	Hydroxyl-β-cyclodextrin	Chloroform: methanol (2:1)		In vitro study for wound healing application	^102^
15	PLA-Hyperbranched polyglycerol	Curcumin	Chloroform and methanol		In vitro study for wound healing application	^63^
16	PLA/CS	Curcumin	Trifluoro acetic acid: DCM (80:20)		In vitro and in vivo wound healing study	^103^
17	PLA/PEG	Curcumin	DMF and Chloroform (1:9)		In vitro study for evaluating potential biomedical applications	^104^
18	PLGA	Curcumin	Methanol: Chloroform		In vitro evaluation against squamous carcinoma	^105^
19	PLGA/ Mesoporous silica nanoparticle	Curcumin	DMF and DCM (1:4)		In vitro evaluation against breast cancer cells	^106^
20	PVA	Curcumin-β-cyclodextrin	PVA (10% w/v)		In vitro study for evaluating potential biomedical applications	^107^
21	PVA	Curcumin	Acetic acid (20% v/v)		In vitro study for wound healing application	^108^
22	PVA/honey	Curcumin	–		In vitro study for evaluating potential biomedical applications	^59^
23	PVA/cellulose acetate	Curcumin	–		In vitro study for wound healing application	^109^
24	PVA/PCL/Carbopol/ Chitosan	Curcumin/ Buccal fat pad-derived mesenchymal stem cells	Acetic acid, DMF and chloroform		In vitro study for evaluating potential biomedical applications	^110^
25	PVA/PEG/PLA	Curcumin	Chloroform : Acetone (2:1)		In vitro study for wound healing application	^111^
26	PVA/PCL	Curcumin	DMF:DCM (2:1 v/v)		In vitro study for wound healing application	^112^

*DCM*, Dichloromethane *DMF*, Dimethylformamide *IPA*, Isopropyl alcohol *DMA*, Dimethylacetamide.

Despite having several advantages, a significant drawback of PCL is its hydrophobicity, due to which cell attachment decreases and the drug release rate falls quickly after an initial burst.^95^ Thus, using hydrophilic pore generating polymers like Polyethylene glycol (PEG), methoxy Polyethylene glycol, and gelatin can overcome this issue. High vapor pressure solvents like dichloromethane serve the same purpose.^114-116^ A study by Bui et al. showed that the addition of PEG led to a porous nanofiber after being subjected to phosphate buffered saline (PBS). The presence of pores on the surface would enhance its surface area and improve cell attachment. The authors prepared Curcumin-loaded PCL nanofiber mats with 10% (w/w) PEG to examine its potential wound healing assets and had a mean diameter of 680 nm with a diameter range of 300 nm to 1600 nm with the formation of pores. The addition of PEG improved the elongation of the nanofiber mats and had better tensile strength when compared to other nanofibers. A higher wound closure rate report for Curcumin-loaded PCL-PEG nanofibers. Furthermore, the authors report promising anti-inflammatory and anti-bacterial activities against RAW264.7 mouse macrophages and *Staphylococcus aureus*.^90^

Mohammadi and Bahrami^91^ produced PCL nanofibers and gum tragacanth: a natural, cheap, biocompatible, biodegradable, and safe carbohydrate polymer. The nanofibers released Curcumin in a sustained manner over a prolonged period and had excellent biological properties to treat diabetic wounds. The nanofiber diameter increased with the addition of Curcumin, but the change reported was insignificant. However, increasing Curcumin concentration changed the morphology, although without cracks. The contact angle significantly decreased, which implies that the nanofiber became more hydrophilic on the addition of Curcumin. The cell proliferation studies showed a decrease with an increase in Curcumin concentration. The author reported that PCL/GT/Curcumin-3% displayed better cell proliferation than PCL/Curcumin-3%—the outstanding property of gum tragacanth. The percentage release of Curcumin in PCL/Curcumin-3% was 42.6% in 10 days; PCL/gum tragacanth/Curcumin-1% had a gradual drug release, and the percentage release of Curcumin was around 43%. PCL/gum tragacanth/Curcumin-3% nanofibers showed a sustained Curcumin release of 65% in 20 days. The author reports PCL/gum tragacanth/Curcumin-3% as the best way to heal diabetic wounds.

Tetrahydro Curcumin is a major metabolite of Curcumin and a more polar compound than Curcumin. Ravikumar et al. were successful in preparing a beadless, smooth nanofiber patch. The XRD report confirmed the presence of a tiny amount of crystalline Tetrahydro Curcumin on the fiber's surface apart from the entrapped Tetrahydro Curcumin, which might be responsible for its initial burst release from the fiber. The authors report a sustained release following Higuchi's equation, indicating that the drug permeation from the patch followed a diffusion mechanism.^92^

Mohammadi et al. evaluated chrysin-Curcumin-loaded PCL-PEG nanofibers for wound healing. Chrysin is a natural, biologically active flavonoid found in extracts like plant gum, honey and propolis; and reportedly possesses anti-inflammatory and anti-oxidant properties.^117,118^ Increasing chrysin concentration from 5% (w/w) to 10% (w/w) altered the beads' size; on further increasing the concentration to 15% (w/w), an increase in beads with the beads' size remaining the same. The release study indicated that the amount of drug released increases with the amount of the drug encapsulated in the nanofiber; a more significant, faster release of chrysin from nanofibers than that of Curcumin. The in vivo wound closure study suggested that higher doses of both Curcumin and chrysin have higher wound closure rates. However, wound closure was greater than chrysin. Chrysin 5%, Curcumin 10% (w/w) combination-loaded nanofibers showed higher wound closure efficacy when compared to other combinations.^93^

In a study by Sedghi et al. a Zn-Curcumin complex resides in a coaxial nanofiber. Its core contains the complex and PCL, and its shell comprises carboxymethyl chitosan, PVA, and graphene oxide. Due to its excellent biological activity, the chitosan derivative promotes biocompatibility, adhesivity, and anti-microbial activity.^119^ However, the PVA blend can improve the electrospinning quality.^120^ The addition of graphene oxide improves the fibers' mechanical strength and accelerates cellular proliferation and differentiation. The fibers prepared using a coaxial spinneret had an inner diameter of 0.9mm and an outer diameter of 1.3mm. The XRD indicated the absence of the Zn-Curcumin complex from the shell. A higher fibroblast cell attachment to the surface of the nanofiber suggests better biocompatibility and its ability to provide good cell proliferation. Osteoblastic performance assays returned good results relating to the complex. The release profile displayed a burst followed by a controlled release. The authors reported that the fibers showed a cumulative release of 94% and an initial burst release amount of around 49%. The study suggests the potential application of Zn-Curcumin loaded nanofiber scaffolds for efficient bone tissue repairing.^94^

Guo et al.^95^ developed Curcumin-loaded PCL-PEG nanofiber for potential applications in cancer therapy. To prepare an electrospinning polymer solution, the authors synthesized a co-polymer (PCL-PEG-PCL, PCEC). The morphology was smooth, with no drug crystals on the surface and observed an initial burst followed by a slow release of the drug, with the release rate increasing with an increase in the concentration of Curcumin in the nanofiber. Also reported was an increase in the tendency of cell inhibition against glioma 9L cells. The obtained results indicate that the nanofiber mats are an effective drug delivery system for post-operative therapy of glioma tumors.

Sridhar et al. performed an in-vitro evaluation of Curcumin and natural extract-loaded nanofibers to check its efficacy for treating lung and breast cancer. The base polymer was PCL, and the natural extracts included *Azadirachta indica* (neem) and aloe vera. Literature shows that neem extract possesses anti-cancer and anti-oxidant properties.^121-123^ A study has shown that PCL with neem extract has potential in wound dressing and skin reconstitution.^124^ Aloe vera retains water and is advantageous in healing infected skin.^125^^,^^126^ The extracts on incorporation with Curcumin led to an increase in the average diameter of the nanofiber when compared to plain PCL nanofiber. The aloe vera extract improved the mechanical properties of the nanofiber, while the neem extract and Curcumin proved otherwise. Curcumin-neem/PCL reported an encapsulation efficiency of 83%, while Curcumin-aloe vera/PCL nanofiber reported an encapsulation efficiency of 77%. The release profile showed a sustained release following Higuchi model kinetics. The in-vitro cancer cell viability tests showed that the Curcumin-aloe vera combination reduced cell viability against A459 cell lines to 18% and 35% against MCF-7 cells. Due to its inhibiting effects, mechanical properties, and sustained drug release, the authors suggest that the formulation be delivered locally via drug-eluting stents or implants.^96^ Boroumand et al. designed another drug delivery system using PCL/PEG nanofibers to achieve a sustained and prolonged release of Curcumin in the peritoneum and pelvic cavity of a mouse model of endometriosis^97^ and obtained similar results.

PCL is a biodegradable, biocompatible, non-immunogenic, and non-toxic polymer, making it a potential material for drug delivery, wound dressing patches, and scaffolds for tissue engineering purposes. However, the cell attachment of PCL reduces due to its hydrophobic nature; and as a result, the wound closure rate is low. By adding a hydrophilic pore generating polymer such as PEG to PCL, the studies indicated a higher wound closure rate when using a combination of a polymer compared to using it individually. Curcumin concentration plays a vital role in cell cytotoxicity and should vary according to the required application. The PCL-PEG composite can also be used as a delivery vehicle against cancer due to its enhanced properties as projected by its activity against glioma, endometriosis, and lung and breast cancer.

### Polylactic Acid (PLA)

Literature shows substantial work to develop Curcumin-loaded PLA nanofibers (Table II) for wound healing and cancer treatment. PLA is a polymer with excellent mechanical properties, biodegradability, and biocompatibility in the human body, making it ideal for biomedical applications, and used in surgical sutures and bio-implants.^62^ PLA nanofiber dressing also has potential in regenerative medicine. PLA/Curcumin nanofibers have shown good blood compatibility and wound healing properties.^63^

In a study by Pankongadisak et al. the solvent mixture was dichloromethane and dimethylformamaide. Curcumin being lipophilic dissolved easily in the PLLA/dichloromethane/dimethylformamide solution.^101^ More minor diameter results in a high surface-to-volume ratio, improving cell attachment and proliferation, making it suitable for wound dressing application.^127^ The release kinetics showed a burst followed by a controlled release, and the amount released was proportional to the amount incorporated.^101^

In a study done by Bharathi et al. with optimized process parameters and a Curcumin concentration of 11% encapsulated in PLA/chitosan polymer, the system showed an improved anti-oxidant property, and the in-vitro cytotoxic results showed no toxicity on L-929 fibroblast (cell line from the subcutaneous connective tissue of mouse). The in-vivo wound healing study showed an increased healing rate, suggesting the potential of Curcumin loaded PLA/chitosan nanofiber for wound healing application.^103^

Mai et al. fabricated Curcumin-loaded PLA nanofibers with nanoscale diameter dimensions and increased drug loading capacity for potential biomedical applications.^98^ A few months later, a similar study by Thangaraju et al. was published. The authors fabricated a Curcumin-loaded PLLA scaffold and reported controlled drug release. The authors reported the potential application of Curcumin-loaded PLLA nanofiber for drug delivery observing parameters such as water uptake, percentage porosity, morphology, cytotoxicity and in-vitro drug release.^99^ The in-vivo biological assay of the nanofibers was studied by Thuy et al. the following year by developing a nanofiber patch for healing wounds. The estimated mean diameter of Curcumin-loaded PLA nanofibers was 562nm with a range of 300-1200nm with pores on the surface of the nanofiber. The use of a mixture of volatile solvent dichloromethane and a non-volatile solvent N, N-dimethylacetamide might be the reason for the formation of pores on the surface of the nanofiber.^100^

Malathi et al.^105^ reported the effectiveness of Curcumin-loaded Poly(L-lactic-co-glycolic) acid (PLGA) nanofiber for the treatment of squamous carcinoma**.** The addition of glycolic acid to PLA increased its hydrophilicity and improved the cell attachment of the nanofiber. An average diameter of 100-300 nm was reported and had a high yield, and the drug encapsulation efficiency was also high. The release kinetics followed a non-fickian model, and the authors reported a sustained release of Curcumin with no initial burst. The cell viability report suggests that Curcumin-loaded PLGA nanofibers successfully arrested the growth of cancer cells.^105^

A recent study by Mohebian et al. worked on developing an implantable drug delivery device to treat tissue defects after tumor resection. The authors used Curcumin as an anti-tumor agent and encapsulated it in mesoporous silica nanoparticles embedded in PLGA via blending electrospinning. Mesoporous silica nanoparticles can improve bioavailability, and with their small size, they can accumulate at the tumor site caused by enhanced permeation and retention effects. The authors report a sustained and prolonged release behavior along with higher in-vitro cytotoxicity and efficient prevention of tumor metastasis when compared to Curcumin-loaded mesoporous silica nanoparticle or Curcumin-loaded PLGA nanofiber.^106^

Zhang et al. suggested that adding PEG to PLA achieves a faster release profile^128^ to help inhibit the growth of bacteria in and around a wound and can be used for wound dressing applications. Wang et al. fabricated a Curcumin-loaded PLA/PEG composite nanofiber. The authors reported that a decrease in the weight ratio of PEG: PLA changed the composite nanofiber from smooth to porous, and the pore structures were evident for a weight ratio of 1:7. However, the hydrophobicity of the composite nanofiber increased with a decrease in weight ratio. A decrease in weight ratio results in an increase in cumulative Curcumin release due to the appearance of pores. A burst release of Curcumin prevented the presence of pores and better control over the release of the drug.^104^ Govindarajan et al. performed a similar study where hyperbranched polyglycerol was used instead of PEG. The nanofiber exhibits high hydrophilicity and better cell viability, adhesion, and proliferation than Curcumin-loaded PLA nanofiber, and the authors report the same. The authors report a higher release of Curcumin than in Curcumin-loaded PLA, and the wound healing rate was also faster than the latter.^63^

A study by Zeynep and Tamer showed the efficacy of core-shell nanofibers with a core of Curcumin and hydroxyl-β-cyclodextrin and shell of PLA. The authors reported that the fabricated nanofiber showed enhanced solubility property than Curcumin-loaded PLA, resulting in more release than the latter in acidic and neutral environments. Hydroxyl-beta-cyclodextrin improved the solubility of Curcumin in an aqueous solution, and the presence of a core resulted in a slow release of Curcumin. The study states that the core-shell nanofiber structure could provide a steady release and high water solubility for hydrophobic drugs.^102^

PLA is a biodegradable, biocompatible, and non-toxic polymer possessing excellent mechanical properties, which finds its applicability in drug delivery, tissue engineering, fabricating surgical sutures, and regenerative medicine applications. Curcumin-loaded PLA nanofibers have exhibited good wound healing properties. The fibers' hydrophilicity was improved by blending hydrophilic polymers such as glycolic acid, hyperbranched polyglycerol, and PEG. Curcumin-loaded PLA and PLGA nanofibers are promising materials for drug delivery and have shown their efficacy in-vitro against carcinoma and breast cancer.

### Polyvinyl Alcohol (PVA)

Polyvinyl alcohol (PVA) is a non-toxic, water-soluble, biodegradable, and biocompatible synthetic polymer, approved for use in medical applications (Table II), including surgical threads, transdermal patches, preparation of hydrogels, and immediate and sustained drug release formulations. Furthermore, PVA nanofibers are potential wound dressers due to their hydrogel formation properties and ability to control drug release.^59^ Other factors that contribute to its application are its soft consistency, transparency, low interfacial tension, and permeability to small molecules.^60^

Xiao et al. published the first work on Curcumin-loaded PVA nanofibers. The authors prepared and loaded fibers with Curcumin and Curcumin-β-Cyclodextrin complex and reported the presence of crystalline Curcumin while the Curcumin-Cyclodextrin complex lined the fibers. Also, the authors report a reduction in diameter with increased Curcumin concentration, although otherwise when the Curcumin-Cyclodextrin complex concentration increases. Furthermore, the thermal stability of Curcumin improved. A diffusion-controlled release mechanism; and sustained drug release for both Curcumin-loaded PVA and Curcumin-Cyclodextrin-loaded PVA nanofibers took place. The enhanced drug stability and solubility make the Curcumin loaded fibers potential candidates for drug delivery and wound dressing.^107^

However, significant work has taken place recently. In a study by Mahmud et al. Curcumin-loaded PVA nanofibers—cross-linked through heat and UV treatment—were synthesized for biomedical applications. Crosslinking improved the swelling ratio and stability of the samples. The authors reported a controlled Curcumin release with an initial burst release from the fiber, and the predicted mechanism of release was both diffusion and erosion of the matrix. The results indicate the release rate could reduce by 20% in the case of heat cross-linking and 9% in UV cross-linking compared to non-cross-linked nanofibers. In addition, the Curcumin-loaded PVA nanofibers demonstrated an excellent anti-bacterial property, killing 100% of bacteria—both gram-positive and gram-negative—within 6 hours. The authors state that Curcumin-loaded cross-linked PVA nanofibers could be a potential candidate for wound dressing applications.^108^

Abdus et al. reported the possible use of Curcumin-loaded PVA-honey nanofibrous mats in wound dressing and tissue engineering. Honey demonstrates anti-microbial action and activity against inflammation, cell reinforcement, and wound recuperation—enabling its application in wound dressing.^129,130^ The study by Abdus et al. focused on exploring the combined medicinal properties of honey and Curcumin extracts. The Curcumin-loaded PVA-honey nanofiber mats exhibited enhanced moisture management properties. In addition, the authors report an inhibition zone in the range of 29-38mm. Due to these properties of the nanofibrous mats, they could be potential wound dressings.^59^

Mrunalini et al. synthesized three types of layered mats of electrospun nanofibers—a layer of Curcumin-honey-loaded electrospun PVA nanofibers sandwiched in-between layers of Curcumin-loaded cellulose acetate nanofibers—and the other two types are the same except for the middle layer. One type contained Curcumin-loaded PVA nanofibers, and the other contained honey-loaded PVA nanofibers. The multi-layered assembly assists in reducing the release of honey from the middle layer, implying a small concentration of honey is delivered to localized wounds, reducing systemic toxicity offered by high concentrations.^131^ The authors reported that honey and Curcumin fibres in the middle showed almost identical efficacies in their anti-oxidant, anti-microbial activity, and water absorbency. The improved water uptake and quick absorbance are due to cellulose acetate and PVA. The authors report that the multi-layered architecture controlled the transmission of moisture rate, absorbed pus and provided anti-microbial activity against common infections; and thus, the mats can be an effective wound dressing material.^109^

In a study by Golchin et al. the authors prepared a nanofibrous composite scaffold of Carbopol, PVA, PCL, and chitosan. They reported that a concurrent delivery of Curcumin incorporated scaffold and buccal fat pad-derived mesenchymal stem cells showed higher wound healing efficacy in a full-thickness skin wound. The author used hydrophilic polymers such as carbopol and chitosan for fabricating the scaffold and PVA and PCL to improve the fiber properties. The prepared scaffold had good water and protein adsorption, improving cell attachment, growth and viability rate. In addition, the Curcumin release increased with time and a sustained profile was reported. Therefore, Curcumin incorporated scaffold in combination with buccal fat pad-derived mesenchymal stem cells could be a potential candidate for various biomedical applications.^110^

There are studies where PVA is used to enhance the property of another base polymer or improve the drug's stability. Leila et al. synthesized Curcumin-loaded PLA nanofibers cross-linked with PVA/PEG for wound dressing applications. The results indicated an enhancement in the mechanical properties of the fiber (tensile strength, elastic modulus and elongation). Also, due to hydrophilic polymers in the fibers, the nanofiber offered good water absorbance. A porous morphology exists, and drug release takes place in two stages; a burst release of the drug, followed by a constant release rate.^111^ In another study by Seyed et al. PVA was used alongside PCL to fabricate multi-layered nanofibrous structures as an active wound dressing. The authors prepared a three-layered structure with Curcumin-loaded PCL nanofiber on either side and the middle layer comprised of PVA with some amount of Curcumin. The purpose of Curcumin was to provide anti-bacterial and anti-inflammatory activities, so PCL—which provides desirable mechanical properties—was used to encapsulate it, and the purpose of PVA was to improve the absorbance of the exudates. Furthermore, the results showed that the addition of the PVA layer increased absorbability by three times, indicating its efficiency in absorbing exudates. The anti-bacterial test revealed that Curcumin (16% w/w) killed all gram-positive and gram-negative bacteria within 48 hours and is an optimum concentration concerning anti-bacterial activity.^112^ Thus it can be concluded that PVA is an excellent polymer to prepare nanofibers either as a base for a delivery vehicle or to enhance the property of another base polymer for tissue engineering and wound dressing applications.

PVA is a biocompatible, biodegradable, non-toxic, hydrophilic polymer and has hydrogel-forming properties enabling it to have better control over the release of a drug. Also, PVA offers permeability to small molecules, has low interfacial tension, and offers soft consistency and transparency, making it suitable for potential wound dressing applications.

### Miscellaneous Polymers

Other biocompatible and biodegradable synthetic polymers apart from PCL, PLA, and PVA have been used to fabricate electrospun nanofibers to deliver Curcumin (Table III). Nithya et al. prepared Curcumin-loaded Poly(2-hydroxyethyl methacrylate) (pHEMA) for wound healing applications. pHEMA is a biodegradable and biocompatible polymer and a carrier for drug delivery. It has hydrogel-forming abilities, making it an appropriate material for tissue engineering scaffolds due to its mechanical and good mass transfer properties. The authors reported a sustained and controlled release of Curcumin which proved to be efficient against infectious diseases caused by multi-drug-resistant organisms like MRSA (Methicillin-resistant *staphylococcus aureus*).^132^

**Table III:** Scientific Evidence for Potential Application of Curcumin Nanofibrous Drug Delivery Using Miscellaneous Polymers

S. no.	Polymer	Drug(s)	Solvent	Remarks	Reference
1	pHEMA	Curcumin	Ethanol and water (4:1)	In-vitro study for wound healing application	^132^
2	Polyurethane	Curcumin	1,1,1,6,6,6-hexafluoroisopropanol (HFIP)	In-vitro evaluation of anti-bacterial activity	^133^
3	Polyurethane	Curcumin	DMF	In-vitro study for wound healing application	^134^
4	PHB	Curcumin	Chloroform and DMF	In-vitro study for wound healing application	^135^
5	PHBV	Curcumin	Chloroform and DMF (50:50 v/v)	In-vitro study for wound healing application	^136^
6	PBAT	Curcumin and 5-FU	DMF and DCM	In-vitro evaluation for colorectal cancer	^137^
7	CA	Curcumin	Acetone and DMA (2:1 v/v)	In-vitro study for wound healing application	^138^
8	CA-PVP	Curcumin	Acetone and water	In-vitro study for wound healing application	^139^
9	Chitosan-Xanthan	Curcumin	Formic acid	In-vitro evaluation of hydrophobic bioactive delivery carrier	^140^
10	Chitosan-Xanthan	Curcumin	Formic acid	In-vitro permeability evaluation of drug across Caco-2 cells	^141^
11	Chitosan-Zein silk	Curcumin	Formic acid	In-vitro study for wound healing application	^142^
12	Bombyx mori silk	Curcumin	-	In-vitro evaluation for potential drug carrier	^143^
13	Almond gum-PVP	Curcumin-β-cyclodextrin	Ethanol and water	In-vitro evaluation in simulated saliva and gastrointestinal conditions	^144^

*DCM*, Dichloromethane *DMF*, Dimethylformamide *DMA*, Dimethylacetamide.

Polyurethane (PU) is a polymer with good barrier properties, oxygen permeability and biocompatibility, making it worthy for wound dressing applications. Shababdoust et al. published a study in which PU/PCL (2000 and 530 Da) were synthesized and analyzed for their potential application in wound dressing. The authors used hexamethylene diisocyanate as a polymerizing agent, PCL as biocompatible polyol and butanediol as a chain extender. The results showed that PU2000 was more hydrophilic compared to PU530. The release profile showed almost the same pattern for PU2000 and PU530—a burst followed by a controlled release. The authors suggested that PU2000 with 10% Curcumin concentration is a good candidate for wound dressing.^133^ A similar study by Nesrin and Nehir (2019) demonstrated the efficacy of Curcumin-loaded PU nanofibers for wound healing applications.^134^

Polyhydroxybutyrate (PHB) is a polyalkanoate, a class of aliphatic polyesters produced by bacteria during unbalanced growth conditions. It has excellent biocompatibility and biodegradability, and hence a good candidate for biomedical applications. Ghavami et al. designed PHB nanofibrous mats containing Curcumin, and the release profile showed an initial burst followed by sustained release of Curcumin, and percentage release increased for a higher Curcumin concentration. The anti-bacterial test showed the efficiency against gram-positive and gram-negative bacteria. The authors mentioned that a Curcumin concentration of 3% (w/w) displayed good anti-bacterial properties. This concentration exhibited cytotoxicity, and cell adhesion with concentration.^135^ In another study, Poly(3-hydroxybutyrate-co-3-hydroxyvalerate) (PHBV) acted as a delivery vehicle for Curcumin. PHBV is more rigid and elastic when compared to PHB and also has a lower melting point in addition to biodegradability and biocompatibility.^145^ Gozde et al. reported that increased Curcumin concentration reduced the nanofiber's ultimate tensile strength and elasticity, with a sustained Curcumin release, and the cumulative release and release time increased with Curcumin concentration. In addition, the incorporation of 0.5% (w/v) of Curcumin in the PHBV nanofiber increased cell attachment and proliferation and improved pharmacological properties.^136^

Poly (butylene adipate-co-terephthalate) (PBAT)—a synthetic polymer that is biodegradable, non-toxic, non-mutagenic, and has good flexibility and undergoes faster degradation—is proven to have no harmful effect on the environment and human health. Moreover, it allows adjustments in its structure and offers flexibility in various applications. Jaleh et al. produced electrospun PBAT loaded with 5-FU and Curcumin to conduct an in-vitro study to evaluate the efficacy of co-delivery and compare it with single drug delivery against colorectal cancer. Increasing the drug concentration reportedly increased the fiber diameter, which might be due to the increase in viscosity on the addition of the drug. Authors reported that the PBAT polymer showed enhanced mechanical properties like elongation percentage, Young's modulus and tensile strength showing its high draw ability and extensibility. The release profile followed a burst release followed by a sustained release, which was beneficial for the treatment. The cytotoxicity results indicated that the drug combination exhibits higher toxicity when compared to free drug solution or single drug-loaded nanofiber. The study indicates that the system could be used in drug-eluting stents as it increases the efficacy of 5-FU and decreases the possibility of any systemic effects.^137^

Cellulose acetate (CA) is the acetate ester of cellulose, the primary structure of the cell wall of green plants. It is biodegradable, non-toxic and is one of the most common biopolymers on earth. It is lightweight, easy to process, recyclable and has good mechanical and barrier properties.^146^ A study by Suwantong et al. on Curcumin-loaded electrospun CA nanofiber reported smooth morphology and no Curcumin aggregates on the surface. A sustained release profile was reported, with increased amounts released with increased Curcumin concentration.^138^ Petya et al. developed novel electrospun material from CA and Polyvinylpyrrolidone (PVP) to deliver Curcumin using the dual spinneret electrospinning technique. PVP is a water-soluble polymer, and a combination of CA and PVP nanofiber mats had enhanced hydrophilicity. The incorporated Curcumin was in an amorphous state, which helps enhance the bioavailability. The Curcumin release was compared with Curcumin/CA, Curcumin/PVP, Curcumin/CA+PVP, Curcumin/CA+ Curcumin/PVP. Curcumin released from Curcumin/CA+ Curcumin/PVP was more significant than the other three nanofiber mats. Curcumin/CA+ Curcumin/PVP possessed more significant anti-bacterial activity, killing all bacteria within 4 hrs. The results suggest that the prepared electrospun nanofibers are potential applications for wound dressing.^139^

Chitosan is a natural cationic polysaccharide known for its biocompatibility, biodegradability, mucoadhesive, and drug absorption enhancement. Elhamalsadat et al. investigated Xanthan-Chitosan nanofibers as a potential carrier of Curcumin in various pH media. Xanthan gum is an anionic polysaccharide used as an encapsulating matrix that improves the stability of chitosan by forming a stable complex.^141^ The authors reported stable nanofibers but the adhesion property of the fibers reduced on the addition of Curcumin due to its hydrophobicity. The release of Curcumin in acidic media was lower when compared to neutral media. The author concluded that the prepared nanofibers have high encapsulation efficiency, good physical stability, and long-term pH stimulated release properties.^140^ In the same year, Adele et al. studied the in-vitro permeability enhancement of Curcumin across Caco-2 cells monolayers using electrospun xanthan—chitosan nanofibers. The authors reported that the prepared nanofibers attained stability in an aqueous Hanks' balanced salt solution (HBSS) at pH 6.5 and 7.4 with a sustained release of Curcumin without any burst effect. The release of Curcumin lasted for only 4 hours, beyond which there was no significant cumulative release. After 24 hours of Caco-2 cells exposure, cell viability of 80-90% were achieved.^141^ A recent study by Kohal et al. studied the potential application of Curcumin-loaded zein-silk fibroin-chitosan nanofibers as an active wound dressing mat. Zein is a protein found in corn and is composed of amino acids. Due to its biocompatibility, biodegradability, flexibility, anti-oxidant activity, and resistance to microorganisms have various biomedical applications. Silk fibroin is a natural protein produced by Bombyx mori and a core comprising fibroin protein and a glue-like coating composed of sericin protein. It also has excellent mechanical strength, biodegradability, non-cytotoxic, non-carcinogenic, and non-inflammatory characteristics. Smooth and uniform nanofibers enhanced Curcumin's mechanical property and thermal stability. The release study showed a burst followed by a controlled release. The cell proliferation and attachment improved, and the cytotoxicity test revealed the nanofibers being non-toxic and biocompatible.^142^

Thangaraju et al. dealt solely with Bombyx mori silk nanofibers and evaluated Curcumin-loaded ones with an average diameter of 50-200nm for Curcumin-loaded silk nanofibers. The XRD and thermal analysis confirmed the presence of amorphous Curcumin in the fiber. The high porosity and high water uptake abilities make it suitable for drug delivery. The release kinetics followed a diffusion model, and the release rate was controlled and sustained after an initial rapid release which might be due to the presence of loosely bound Curcumin on the fiber surface. The biocompatibility, good water uptake abilities and sustained drug release make the silk nanofiber a suitable drug carrier.^143^

Another natural polymer is almond gum, a soluble polysaccharide and a viable choice for compound stability. In the study by Atefe and Ali, Curcumin and Curcumin-β-cyclodextrin complex were encapsulated in almond gum/ PVP nanofibers. The author reported higher concentration of Curcumin caused beads to form on the fiber surface. A Curcumin concentration of 1% or 2% produced bead free nanofiber. Furthermore, Curcumin-β-cyclodextrin complex concentrations greater than 4% lead to bead formation. The loading efficiency of nanofibers containing the complex was more significant than the samples containing only Curcumin, and increasing the complex concentration increased the loading efficiency. The authors evaluated the release profile in two conditions: stimulated saliva and stimulated gastrointestinal conditions. The cumulative release was significant for the complex in both conditions because Curcumin's solubility and stability increase when it forms a complex with Curcumin-β-cyclodextrin.^144^

Apart from PCL, PLA and PVA, many other polymers have produced nanofibers for biomedical applications. Amongst synthetic polymers, PU offers excellent gas permeability and barrier properties, making it an optimal choice for wound dressing applications, whereas PHB and PHBV offer good biocompatibility and biodegradability, provide good mechanical provides, and enhance Curcumin cell attachment and proliferation. Hence the polymers are best suited for drug delivery and wound dressing applications. PBAT offers flexibility to alter its chemical structure in applications, including drug delivery. pHEMA is another polymer having hydrogel-forming abilities, hence offering reasonable control over the release of the drug.

Chitosan is a popular choice due to its enhanced drug absorption capabilities, biocompatibility, biodegradability, mucoadhesive. Therefore it is an excellent candidate for drug delivery applications. Chitosan spun into nanofibers with another polymer such as xanthan gum, zein, or/and silk fibroin as chitosan has poor stability. Nanofibers spun out of silk fibroin showed good drug delivery abilities. Cellulose acetate is a biodegradable, biocompatible, and most abundantly found natural polymer. It has an excellent barrier and mechanical property, and therefore a good candidate for wound dressing applications.

## Conclusion and Prospects

Allopathic medicines, although potent, demand a relatively higher concentration of drugs to be consumed, of which only a tiny portion will reach the target area. This mode of drug delivery renders adverse effects on other parts of the body and puts the kidneys and liver under stress.

The drug is released only at the target site using nanofibers, thus protecting the kidneys and liver. The use of herbal drugs has no unwanted side effects, and incorporation in nanofibers will improve the solubility of the drug and its bioavailability by controlling the release rate of the drug.

The literature presents various combinations of polymers used to synthesize Curcumin-loaded nanofibers for medical applications. Amongst synthetic polymers, PCL and PVA are promising candidates as they have shown good results in cancer treatment, tissue engineering and wound healing applications. In natural polymers, chitosan is a promising candidate. Though natural polymers appeal over synthetic due to their availability and subsequent low manufacturing cost, the drug's stability is poor. Thus natural polymers can be blended with a synthetic polymer in adequate proportions to make the drug stable and keep the costs low.

Studies have mainly focused on delivering Curcumin by nanofiber for wound healing application. However, extensive work is exigency on Curcumin or a combination of Curcumin with another drug for cancer therapy. Future work should focus on synthesizing nanofibers using forcespinning as it aids in mass production, including various biomaterials to test on human cancer cells. Also, searching for an easier and more comfortable means of drug delivery, such as oral means, will be less painful and more acceptable for the general public. The inclusion of artificial intelligence to develop nanofiber-drug combinations—substantiated with release concentration, type of release, efficacy—against various tumor types is an exciting prospect.

Despite proving its potency in various medical applications that are of great interest, the commercial use of Curcumin to address severe medical conditions is still unrealized. Such a situation could be due to many reasons, one of which is the hypocritical change of heart, especially when the illness is life-threatening. Another is its bioavailability and cost, for which nanotechnology gives a befitting reply. However, "the elephant in the room" is trust when the stakes are high! To overcome this barrier, we urge more extensive research to establish trust not only in the ordinary person but, more importantly, in scientists and medical practitioners who ultimately are the ones responsible for the commercial use of this ancient medicine.
